# Ox-LDL Promotes Migration and Adhesion of Bone Marrow-Derived Mesenchymal Stem Cells via Regulation of MCP-1 Expression

**DOI:** 10.1155/2013/691023

**Published:** 2013-07-17

**Authors:** Fenxi Zhang, Congrui Wang, Huaibin Wang, Ming Lu, Yonghai Li, Huigen Feng, Juntang Lin, Zhiqing Yuan, Xianwei Wang

**Affiliations:** ^1^Department of Anatomy, Sanquan College, Xinxiang Medical University, Xinxiang, Henan 453003, China; ^2^Stem Cell and Biotherapy Technology Research Center, College of Life Science and Technology, Xinxiang Medical University, Xinxiang, Henan 453003, China; ^3^Division of Cardiology, University of Arkansas for Medical Sciences, Little Rock, AR 72205, USA

## Abstract

Bone marrow-derived mesenchymal stem cells (bmMSCs) are the most important cell source for stem cell transplant therapy. The migration capacity of MSCs is one of the determinants of the efficiency of MSC-based transplant therapy. Our recent study has shown that low concentrations of oxidized low-density lipoprotein (ox-LDL) can stimulate proliferation of bmMSCs. In this study, we investigated the effects of ox-LDL on bmMSC migration and adhesion, as well as the related mechanisms. Our results show that transmigration rates of bmMSCs and cell-cell adhesion between bmMSCs and monocytes are significantly increased by treatments with ox-LDL in a dose- and time-dependent manner. Expressions of ICAM-1, PECAM-1, and VCAM-1 as well as the levels of intracellular Ca^2+^ are also markedly increased by ox-LDL in a dose-dependent manner. Cytoskeleton analysis shows that ox-LDL treatment benefits to spreading of bmMSCs and organization of F-actin fibers after being plated for 6 hours. More interestingly, treatments with ox-LDL also markedly increase expressions of LOX-1, MCP-1, and TGF-**β**; however, LOX-1 antibody and MCP-1 shRNA markedly inhibit ox-LDL-induced migration and adhesion of bmMSCs, which suggests that ox-LDL-induced bmMSC migration and adhesion are dependent on LOX-1 activation and MCP-1 expression.

## 1. Introduction

 Mesenchymal stem cells (MSCs) are multipotent progenitor cells that can differentiate into several types of cells, including osteocytes, adipocytes, chondrocytes, endothelial cells, cardiomyocytes, and neurons when exposed to appropriate conditions [1, 2]. Bone-marrow derived MSCs (bmMSCs) are the most widely used MSCs in tissue regenerative medicine. It has been reported that bmMSC transplantation has therapeutic benefits to many kinds of diseases such as Alzheimer's disease, heart infarction, stroke, and rheumatoid arthritis [3–6]. The migration capacity of bmMSCs is the most important determinant of the efficiency of bmMSC transplant therapy. It has been shown that less than 1.5% bmMSCs can reach the injured tissues after intracoronary injection [7]. The low homing rate of bmMSCs after transplantation severely limits their clinical uses. Another limitation of bmMSC transplant therapy is the poor viability of bmMSCs after transplantation [8]. Cell adhesion is a prerequisite for the survival of the transplanted bmMSCs and is also responsible for bmMSC migration [8–10]. 

 Intracellular Ca^2+^ is an important regulator of cell adhesion and migration. The increase of intracellular Ca^2+^ is required for integrin-mediated cell adhesion [11, 12]. Intracellular Ca^2+^ also participates in regulating organization of cytoskeleton [13]. The dynamic rearrangement of cytoskeleton is required for cell adhesion and migration. Ox-LDL is an important stimulator for inflammation and cell adhesion. Previous studies have shown that ox-LDL induces migration of monocytes and smooth muscle cells [14]. A recent study from our group showed that LOX-1, a receptor of ox-LDL, is highly expressed in bmMSCs, and its activation by ox-LDL stimulates proliferation of bmMSCs [1]. Actually, LOX-1 itself also serves as an inflammatory and adhesive molecule, and it is involved in migration of leukocytes [15]. 

 Monocyte chemoattractant protein-1 (MCP-1) is an important regulator of the genesis of acute and chronic inflammation. It plays a key role in monocyte activation and recruitment to the injured sites. Previous studies have shown that MCP-1 mediates transmigration of monocytes and THP-1 cells [16]. It has been reported that ox-LDL through activating LOX-1 enhances MCP-1 expression in the cultured chondrocytes, vascular smooth muscle cells, endothelial cells, and macrophages [17–21]. The ox-LDL-mediated MCP-1 upregulation has been involved in expression of adhesion molecules in endothelial cells [19, 20]. Whether ox-LDL affects bmMSC migration and adhesion and MCP-1 expression in bmMSCs has not been examined. In the present study, we investigated the effects of ox-LDL on bmMSC migration and adhesion, as well as their possible mechanisms. 

## 2. Materials and Methods

### 2.1. Materials

 Ox-LDL and Dil-ox-LDL were purchased from Biomedical Technologies, Inc. (Stoughton, MA, USA). Fluo-3/AM, Rhodamine phalloidin, Lipofectamine LTX kit, RNeasy Mini-Kit, SuperScript II 1st-strand DNA synthesis kit and cell tracker were obtained from Invitrogen (Carlsbad, CA, USA). LOX-1 and MCP-1 antibodies were purchased from Abcam (Cambridge, MA, USA); TGF-*β*, ICAM-1, PECAM-1, VCAM-1, and *β*-actin antibodies were purchased from Santa Cruz Biotechnology, (Santa Cruz, CA, USA). MCP-1 shRNA kit was purchased from OriGene Technologies (Rockville, MD, USA). ECL Western-blotting substrate was purchased from Thermo Scientific (Rockford, IL, USA). The PVDF membrane was purchased from GE Healthcare (Pittsburgh, PA, USA). 

### 2.2. Isolation and Culture of bmMSCs

 BmMSCs were isolated and cultured as previously described [1]. Mice (C57BL/6J, 8-week old) were killed by cervical dislocation. The animals were rinsed in 70% ethanol for 20 seconds to make the bodies sterile, and then the limbs were collected by surgery and put in DMEM medium on ice. After cleaning the muscles, the tibia and femur were cut just below both ends of the marrow cavities. The bone marrow was flushed out using DMEM medium in a 10 mL syringe with a 25-gauge needle and collected in a 15 mL tube on ice. After centrifugation, bone marrow was suspended in DMEM by pipetting several times and filtered through a 70 mm filter mesh to remove the bone spicules and cell clumps. The cell density was calculated by cell counting under a microscope. Then, the cells were plated into 100 mm Petri dishes at the densities of 10 × 10^6^/mL in complete DMEM medium with 15% FBS, 2 mM L-glutamine, 100 *μ*g penicillin, and 100 *μ*g streptomycin, and they cultured for 3 h. After 3 h, the nonadherent cells were removed, and the fresh medium was replaced. Thereafter, the medium was replaced every 2 days. A purified population of bmMSCs can be obtained after 3-week cultureing period.

### 2.3. Dil-ox-LDL Uptake Measurement

 The primary and the 3rd-passage bmMSCs were plated in 24-well plates and incubated with 5 *μ*g/mL Dil-ox-LDL in the dark at 37°C for 30 min. Then, the cells were gently washed with PBS for 3 times, and they were imaged with a fluorescent microscope. 

### 2.4. Transwell Migration Assay

 In this study, migration of bmMSCs was measured using Transwell plates (Corning Costar, USA) with 8 *μ*m pore filters. In brief, human umbilical vein endothelial cells (HUVECs) were seeded into the upper inserts of Transwell chamber (4 × 10^4^ cells/well), and they cultured for 24 h. BmMSCs were treated with 0, 5, 10, and 20 *μ*g/mL ox-LDL for 6 h or treated with 10 *μ*g/mL ox-LDL for 0, 3, 6, and 12 h, and then they were washed with PBS. The washed cells (1 × 10^5^) were plated onto HUVECs in the upper inserts of Transwell plates. After 6 h of coculture, the numbers of migrated bmMSCs on the lower side of the filters were counted.

### 2.5. Cell Adhesion Assay

 BmMSCs were plated in 12-well plates. Monocytes were darkly preincubated with cell tracker at 37°C for 30 min and washed with PBS for 3 times. When bmMSCs were nearly 80% confluent, they were incubated with 0, 5, 10, and 20 *μ*g/mL ox-LDL for 6 h. Then, the predyed monocytes (2 × 10^4^) were seeded onto bmMSCs (washed with PBS) and coincubated for 30 min in the dark. And then, the cells were gently washed with PBS for 3 times and randomly imaged with a fluorescence microscope.

### 2.6. RT-PCR Assay

In this study, LOX-1 expression in bmMSCs was measured by RT-PCR assay. In brief, total RNA was isolated from bmMSCs using RNeasy Mini-Kits according to the kit's instructions; 1 *μ*g RNA was applied to synthesize cDNA with SuperScript II 1st-strand DNA synthesis kits. PCR assay was performed using a 20 *μ*L reaction volume containing 100 ng cDNA, 10 *μ*L 2× PCR reaction mixture, and 0.5 *μ*M primers. The products were separated by 1.5% agarose gel electrophoresis and visualized by ethidium bromide on a UV transilluminator. The primers for LOX-1 were the following: forward: 5′-GAGCTGCAAACTTTTCAGG-3′, reverse: 5′-CTCTTTCATGCGGCAACAG-3′; the primers for *β*-actin were the following: forward: 5′-TTCTTTGCAGCCCTTCGTTGCCG-3′, reverse: 5′-TGGATGGCTACGTACATGGCTGGG-3′.

### 2.7. Western-Blotting Assay

 Proteins were extracted from bmMSCs and separated by 12% SDS-PAGE. After electrophoresis, proteins were transferred to the PVDF membranes. The membranes were blocked with 5% BSA or 5% no-fat milk (according to the manufacturer's instructions) in TBS-T, and they were then incubated with LOX-1, MCP-1, TGF-*β*, ICAM-1, VCAM-1, PECAM-1, and *β*-actin (1 : 2000) primary antibodies at 4°C overnight. Then, the blots were incubated with HRP-conjugated secondary antibodies (1 : 10000) for 1 h at room temperature. The immunoreactive bands were visualized by enhanced chemiluminescence. 

### 2.8. Immunofluorescence Staining

 Immunostaining was performed using standard protocols. In brief, the bmMSCs grown on 10 mm round coverslips were treated with 0, 5, 10, and 20 *μ*g/mL ox-LDL for 6 h. Then, the cells were fixed with 4% buffered paraformaldehyde for 15 min and treated with 0.1% Triton X-100 for 10 min at room temperature. And then, the cells were blocked with 1% BSA for 1 h and incubated with rabbit anti-mouse ICAM-1, VCAM-1, and PECAM-1 antibodies (1 : 200) for 1 h at room temperature. After washing with PBS, the cells were incubated with TR- or FITC-conjugated duck anti-rabbit secondary antibody (1 : 1000) in the dark. After washing, the cells were mounted on slides using ProlongH Gold antifade reagent with DAPI and imaged with a fluorescence microscope. Fluorescent density of ICAM-1, VCAM-1, and PECAM-1 was measured using Image J 1.34 software in several random fields. The average fluorescent density was calculated from 100 cells of each sample.

### 2.9. Flowcytometry Assay

 In this study, intracellular Ca^2+^ of bmMSCs was measured by flowcytometry assay. Briefly, bmMSCs were plated in 6-well plates and treated with 0, 5, 10, and 20 *μ*g/mL ox-LDL for 6 h. Then, the cells were loaded with 5 *μ*M Fluo-3/AM and darkly incubated for 30 min at 37°C. The cells were collected and washed with PBS for 3 times. The washed cells were resuspended in 500 *μ*L PBS and analyzed with a flowcytometer.

### 2.10. Cytoskeleton Analysis

 BmMSCs were plated in 24-well plates and immediately exposed to ox-LDL. After 6 h exposure, the cells were fixed using with 4% buffered formaldehyde, treated with 0.1% Triton-X-100, and then labeled with 2U Rhodamine phalloidin for 30 min in the dark. After washing for 3 times, fluorescence was imaged with laser-inverted confocal microscope.

### 2.11. MCP-1 shRNA

 BmMSCs were plated in 6-well or 12-well plates. When the cells reached 80% confluence, shRNA was performed using Lipofectamine 2000 in Opti-MEM medium and a CCL2 (MCP-1) shRNA kit including CCL2 shRNA duplexes and noneffective 29-mer scrambled shRNA according to the kit's instruction. 

### 2.12. Statistical Analysis

 Statistical analysis was performed with SPSS 11.5 software. Data were presented as the mean ± standard deviation (SD). Univariate comparisons of means were evaluated using appropriate Student's *t*-tests and/or one-way ANOVA with Tukey's post hoc adjustment for multiple comparisons; *P* < 0.05 was considered a statistically significant difference. 

## 3. Results 

### 3.1. Dil-ox-LDL Uptake and LOX-1 Expression in the Primary and the 3rd-Passage bmMSCs

**Figure 1:**
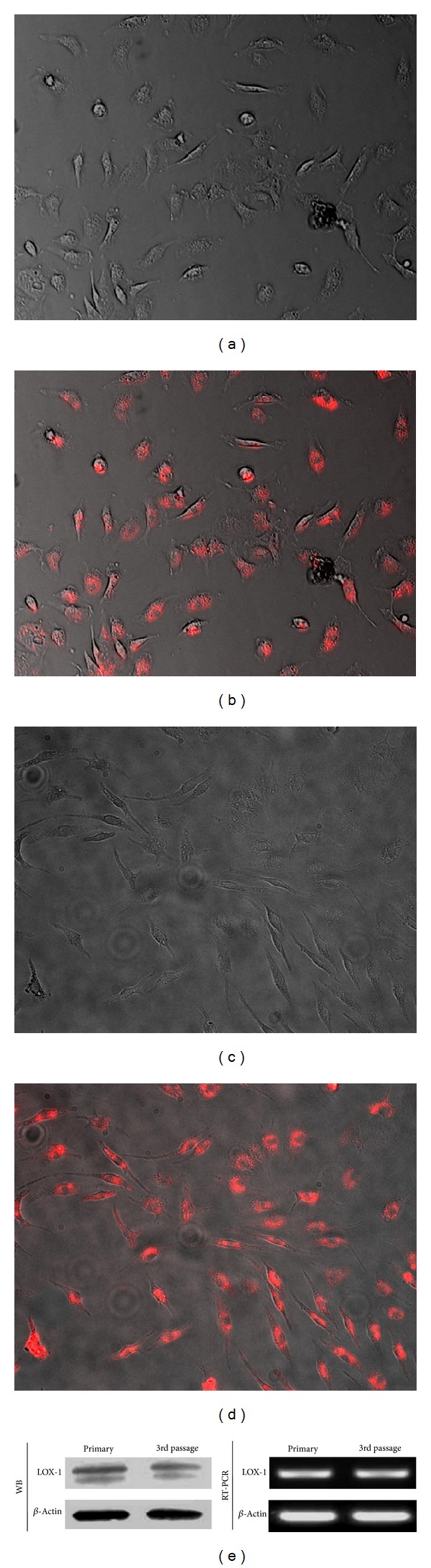
Uptake of Dil-ox-LDL and LOX-1 expression in bmMSCs. (a) Morphology of primary bmMSCs; (b) Dil-ox-LDL uptake in primary bmMSCs; (c) Morphology of the 3rd-passage bmMSCs; (d) Dil-ox-LDL uptake in the 3rd passage bmMSCs; (e) RT-PCR and Western-blotting assays show LOX-1 expression in the primary and the 3rd-passage bmMSCs.

 In a recent study, we had identified the characteristics of bmMSCs and found that the primary bmMSCs have a potential to take up ox-LDL and highly express LOX-1 receptors [1]. In the present study, we observed that the passaged (the 3rd passage) bmMSCs have the same potential to take up ox-LDL and express LOX-1 receptors with the primary bmMSCs (Figure 1). 

### 3.2. Ox-LDL Stimulates Transmigration of bmMSCs in a Dose- and Time-Dependent Manner

**Figure 2:**
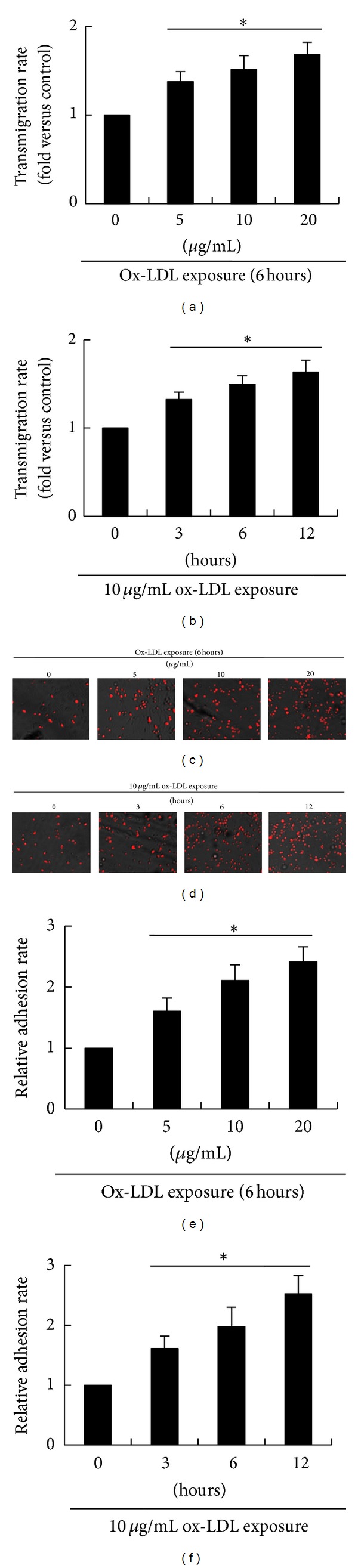
Ox-LDL promotes transmigration of bmMSCs and enhances cell adhesion between bmMSCs (grey color) and monocytes (red color). (a) Transmigration rates of bmMSCs after exposure to 0, 5, 10, and 20 *μ*g/mL ox-LDL for 6 hours; (b) transmigration rates of bmMSCs after exposure to 10 *μ*g/mL for 0, 3, 6, and 12 hours; (c) the merged phase contrast and fluorescence images show adhesion between bmMSCs and monocytes after treatment with 0, 5, 10, and 20 *μ*g/mL ox-LDL for 6 hours; (d) the merged phase contrast and fluorescence images show adhesion between bmMSCs and monocytes after treatment with 10 *μ*g/mL for 0, 3, 6, and 12 hours; (e) the relative adhesive rate of monocytes onto bmMSCs after treatment with 0, 5, 10 and 20 *μ*g/mL ox-LDL for 6 hours; (f) the relative adhesive rate of monocytes onto bmMSCs after treatment with 10 *μ*g/mL ox-LDL for 0, 3, 6, and 12 hours. Bar graphs represent mean ± SD (*n* = 4 per group). *P < 0.01 versus control.

 The migration ability of bmMSCs was measured using a Transwell system. As shown in Figure 2(a), ox-LDL at doses of 5~20 *μ*g/mL significantly increases transmigration rates of bmMSCs (*P* < 0.01) in a dose-dependent manner. From the preliminary data of transmigration of bmMSCs after being exposed to 5~20 *μ*g/mL ox-LDL, we saw that 10 *μ*g/mL ox-LDL exposure caused the medium levels of increase of cell transmigration. So, 10 *μ*g/mL ox-LDL was selected to study the time-dependent transmigration of bmMSCs. When exposed to 10 *μ*g/mL ox-LDL, bmMSCs also exhibit an increased transmigration in a time-dependent manner (Figure 2(b)). 

### 3.3. Ox-LDL Enhances bmMSC Adhesive Ability and Expression of Adhesive Molecules

 It is known that cell adhesion is a critical factor for cell transmigration, and the capacity of cell migration is dependent on expression of adhesive molecules [22]. In this study, adhesive ability of bmMSCs was measured by evaluating cell-cell adhesion between bmMSCs and monocytes. As shown in Figures 2(c)–2(f), the numbers of monocytes adhered to bmMSCs (pretreated with 5 ~ 20 *μ*g/mL ox-LDL) were significantly (*P* < 0.01) increased by treatments with ox-LDL in a dose-dependent manner. When bmMSCs were exposed to 10 *μ*g/mL ox-LDL, the numbers of adhered monocytes were also significantly increased (*P* < 0.01) in a time-dependent manner. 

**Figure 3:**
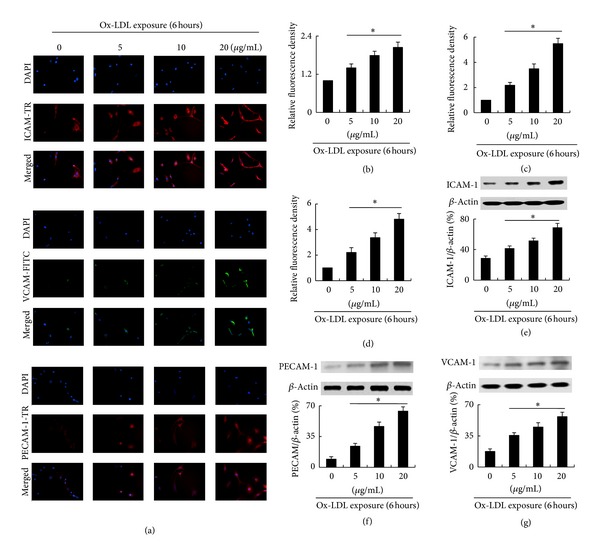
Ox-LDL increases expression of ICAM-1, PECAM-1, and VCAM-1 in a dose-dependent manner in bmMSCs. (a) Immunofluorescence assay shows expression of ICAM-1, PECAM-1 and VACM-1 in bmMSCs exposed to 0, 5, 10 and 20 *μ*g/mL ox-LDL for 6 hour; (b)–(d) Relative fluorescence density of ICAM-1, PECAM-1, and VCAM-1; (e)–(g) Western-blotting assay shows expression of ICAM-1, PECAM-1, and VCAM-1 in bmMSCs exposed to 0, 5, 10, and 20 *μ*g/mL ox-LDL for 6 hours. Bar graphs represent mean ± SD (*n* = 4 per group). *P < 0.01 versus Control.

 Cell-cell adhesion is dependent on expression of adhesive molecules. Our results showed that expression of the adhesive molecules ICAM-1, PECAM-1, and VCAM-1 in bmMSCs was significantly increased (*P* < 0.01) by induction with ox-LDL in a dose-dependent manner (Figure 3). 

### 3.4. Ox-LDL Increases Intracellular Ca^2+^

**Figure 4:**
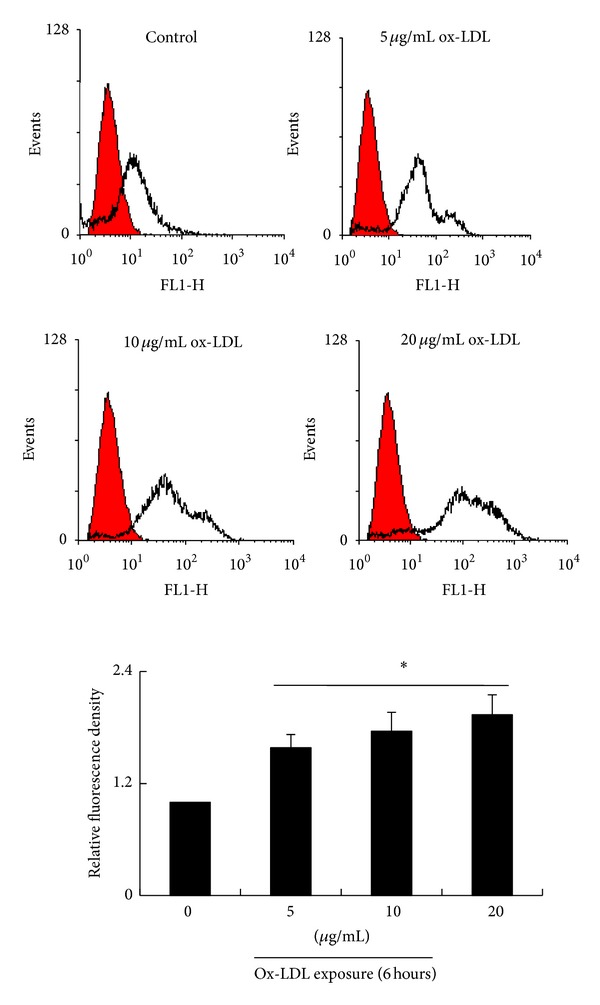
Flowcytometry assay shows the levels of intracellular Ca^2+^ of bmMSCs exposed to 0, 5, 10, and 20 *μ*g/mL ox-LDL for 6 hours. Bar graphs represent mean ± SD (*n* = 4 per group). *P < 0.01 versus control.

 Intracellular Ca^2+^ is an important regulator of cell migration. It has been reported that ox-LDL causes an increase of intracellular Ca^2+^ in other cell lineages such as endothelial cells and smooth muscle cells [23, 24]. In the present study, we also found that ox-LDL (5~20 *μ*g/mL) causes an increase of intracellular Ca^2+^ in bmMSCs in a dose-dependent manner (Figure 4).

### 3.5. Ox-LDL Mediates Reorganization of Cytoskeleton in bmMSCs

**Figure 5:**
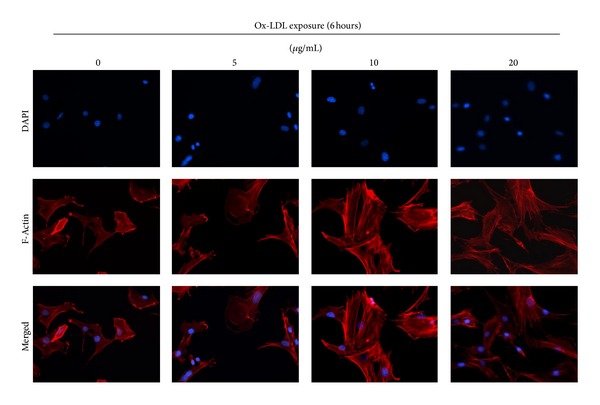
Cytoskeleton (F-actin fibers) organization in bmMSCs after exposure to 0, 5, 10, and 20 *μ*g/mL ox-LDL for 6 hours.

 Cytoskeleton has been known to regulate cell migration and adhesion [25]. In this study, cytoskeleton organization was studied by staining F-actin using Rhodamine phalloidin. Compared with the control, bmMSCs treated with ox-LDL had better spreading and more integrated networks of F-actin filaments (Figure 5).

### 3.6. Ox-LDL Induces Expression of LOX-1, MCP-1, and TGF-*β*

**Figure 6:**
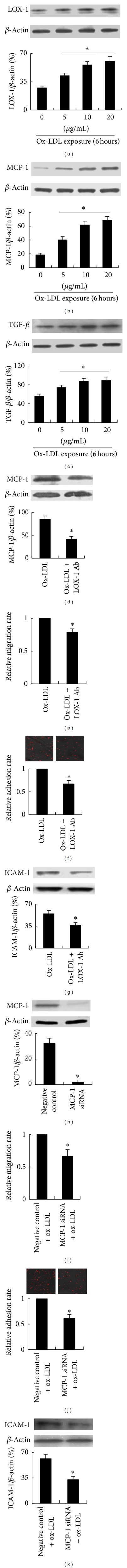
Role of LOX-1 and MCP-1 in ox-LDL-mediated migration and adhesion of bmMSCs. (a)–(c) Western-blotting assay shows LOX-1, MCP-1 and TGF-*β* expression in bmMSCs after exposure to 0, 5, 10, and 20 *μ*g/mL ox-LDL for 6 hours. (d) Western-blotting assay shows that LOX-1 antibody inhibits ox-LDL-induced MCP-1 expression. (e) Transwell assay shows that LOX-1 antibody inhibits ox-LDL-induced transmigration of bmMSCs. (f) LOX-1 antibody inhibits ox-LDL-induced cell adhesion between bmMSCs and monocytes. (g) Western-blotting assay shows that LOX-1 antibody decreases ICAM-1 expression. (h) Western-blotting assay shows MCP-1 expression after transfection of noneffective shRNA and MCP-1 shRNA. (i) MCP-1 knockdown inhibits ox-LDL-induced migration of bmMSCs. (j) MCP-1 knockdown inhibits ox-LDL-induced cell-cell adhesion between monocytes and bmMSCs. (k) MCP-1 knockdown decreases ox-LDL-induced ICAM-1 expression. Bar graphs represent mean ± SD (*n* = 4 per group). *P < 0.01 versus control, ox-LDL treatment, or negative control transfection.

 Our previous study has shown that ox-LDL stimulates LOX-1 expression in bmMSCs [1]. In accordance with the previous study, we observed in this study that ox-LDL (5~20 *μ*g/mL) induces LOX-1 expression in a dose-dependent manner (Figure 6(a)). Furthermore, ox-LDL also increases MCP-1 and TGF-*β* expression in bmMSCs in a dose-dependent manner (Figures 6(b) and 6(c)). 

More importantly, pretreatment with LOX-1 antibody inhibits ox-LDL-induced MCP-1 expression (Figure 6(d)), cell migration (Figure 6(e)), adhesion (Figure 6(f)), and expression of ICAM-1 (Figure 6(g)), PECAM-1, and VCAM-1 (data not shown). These data suggest that ox-LDL-induced adhesion and migration of bmMSCs are at least partially via activation of LOX-1 receptors.

### 3.7. MCP-1 Knockdown Inhibits Ox-LDL-Induced Cell Migration and Adhesion

 To further investigate the role of MCP-1 in ox-LDL-induced bmMSC migration and adhesion, we performed MCP-1 shRNA in bmMSCs. As shown in Figure 6(h), compared with transfection of noneffective scrambled shRNA, MCP-1 shRNA significantly downregulates MCP-1 expression in bmMSCs (*P* < 0.01). More interestingly, MCP-1 knockdown also significantly decreases ox-LDL-induced bmMSC transmigration and adhesion, as well as expression of adhesive molecules (Figures 6(i)–6(k); *P* < 0.01).

## 4. Discussion

 In this study, we for the first time investigated the effects of ox-LDL on migration and adhesion of bmMSCs. We found that treatment with ox-LDL enhances migration and adhesion capacity of bmMSCs. We also observed that treatment with ox-LDL increases intracellular Ca^2+^ and expression of LOX-1, MCP-1, and TGF-*β*, and it facilitates cytoskeleton reorganization. More importantly, use of LOX-1 antibody and knockdown of MCP-1 both significantly inhibit ox-LDL-induced bmMSC migration and adhesion, as well as expression of adhesive molecules. These findings indicate that ox-LDL can promote migration of bmMSCs, which is dependent on LOX-1 activation and MCP-1 expression.

 The migration capacity of bmMSCs is one of the most important determinants of the efficiency of bmMSC-based transplant therapy. It has been reported that the intravenously injected bmMSCs have a steady capacity to migrate back to the bone marrow and home to the injured organs by migrating across the endothelium [26]. But, the homing rates of the injected bmMSCs to the injured tissues are very low (<1.5%) [7]. The low homing rate of bmMSCs would severely affect their therapeutic efficiency in transplant therapy. So, it is necessary to find more effective methods to stimulate migration of bmMSCs. It has been reported that ox-LDL can induce production of inflammatory molecules (MCP-1, IL-6, and adhesive molecules), and subsequently promote migration of macrophages and endothelial cells [27–29]. 

 Cell adhesion is a prerequisite for transmigration of the circulating cells. The first step of the intravenously injected bmMSCs to the injured organs is adhering to the endothelium and overcoming the endothelial barrier. The stable cell adhesion affects cytoskeleton reorganization and actin polymerization, facilitates cell protrusion, and leads to directional cell movement [30]. So, expression of adhesion molecules is critically important for cell migration. It has been reported that PECAM-1 is required for TNF-*α*-induced transmigration of leukocytes [31]. Use of PECAM-1 antibody can inhibit migration of leukocytes [31]. Moreover, cell-cell adhesion is also required for survival of the transplanted bmMSCs in the target organs or tissues. In the present study, we found that low concentrations (5~20 *μ*g/mL) of ox-LDL have potential to stimulate bmMSC migration and adhesion and mediate expression of adhesion molecules (ICAM-1, PECAM-1, and VCAM-1). 

 Calcium ion (Ca^2+^) is a very important cellular secondary messenger, which plays a prominent role in signal transduction and cell physiology. A number of studies have shown that intracellular Ca^2+^ regulates cell adhesion and migration. The increase of intracellular Ca^2+^ is in parallel with an increase of adhesion of lymphocytes, erythrocytes, macrophages, and cancer cells [32–35]. And, increase of intracellular Ca^2+^ can also cause upregulation of adhesive molecules such as ICAM-1, PECAM-1, VCAM-1, and E-selectin [36–39]. Moreover, the expression of adhesion molecules is also required for transmission of calcium [37, 39]. In this study, we also observed that expression of ICAM-1, VCAM-1, and PECAM-1 is in parallel with an increase of intracellular Ca^2+^ in bmMSCs. A study by Cook-Mills et al. showed that the response of intracellular Ca^2+^ to VCAM-1 stimulation is dependent on the activation of NADPH oxidase in endothelial cells [39]. Not surprisingly, as a strong stimulator of NADPH oxidase, ox-LDL can increase expression of adhesive molecules and intracellular Ca^2+^ in bmMSCs. Treatment with Ca^2+^ or Ca^2+^ ionophore A23187 was also observed to stimulate migration of smooth muscle cells; and use of Ca^2+^ entry blocker nicardipine inhibited cell migration of these cells [40]. The calcium-mediated cell migration is dependent on its role in regulating cytoskeletal rearrangement [36]. It is known that the dynamic organization of cytoskeleton is a prerequisite of cell migration. In the present study, treatment with ox-LDL facilitates bmMSC spreading and organization of F-actin fibers. Previous studies reported that the regulation of ox-LDL in actin organization is involved in activation of Rho GTPases and PI3K/Akt pathway [41]. However, some other studies reported that high concentrations of ox-LDL (100 *μ*g/mL) cause disorganization of cytoskeleton and death of smooth muscle cells [42]. In our other ongoing studies, we also observed that high concentrations of ox-LDL (>40 *μ*g/mL) have toxicity to bmMSCs and cause apoptosis of bmMSCs. 

 It has been reported that ox-LDL stimulates cell migration via activation of its receptor LOX-1. Our recent study has shown that LOX-1 is highly expressed in primary bmMSCs. In the present study, we also found that LOX-1 is highly expressed in the passaged bmMSCs. More interestingly, blockade of LOX-1 using LOX-1 antibody significantly inhibits ox-LDL-induced MCP-1 expression, cell adhesion, and migration of bmMSCs. This suggests that ox-LDL-induced bmMSC migration is at least partially via activation of LOX-1. 

 MCP-1 is an important regulator of inflammatory events. Previous studies have shown that ox-LDL via activation of LOX-1 enhances MCP-1 expression in many cell lineages such as human articular chondrocytes, vascular smooth muscle cells, endothelial cells, and macrophages [17–21]. Treatment with exogenous recombinant MCP-1 or increase of endogenous MCP-1 expression can induce transendothelial migration of T cells, monocytes, smooth muscle cells, and adult neural stem cells [43–45]. TGF-*β* is another important factor for cell migration. TGF-*β* stimulates cell migration via regulation of MCP-1 expression [44, 46]. In the present study, we also found that ox-LDL stimulates MCP-1 and TGF-*β* expression in bmMSCs in a dose-dependent manner. More importantly, knockdown of MCP-1 expression significantly inhibits ox-LDL-induced bmMSC transmigration, cell-cell adhesion, and expression of adhesion molecules. These data show that the inflammatory factor MCP-1 plays an important role in ox-LDL-induced bmMSC migration and adhesion.

## 5. Conclusion

 In this study, we investigated the effects of ox-LDL on bmMSC migration and adhesion. Our results show that ox-LDL enhances transmigration and adhesion capacities of bmMSCs, which is mediated by LOX-1 activation and MPC-1 expression. Blockade of LOX-1 receptor using antibody significantly decreases ox-LDL-induced MCP-1 expression and inhibits bmMSC transmigration and adhesion. More importantly, MCP-1 knockdown also significantly inhibits ox-LDL-induced bmMSC transmigration and cell adhesion. These findings indicate that MCP-1 plays an important role in ox-LDL-mediated migration and adhesion of bmMSCs.
